# The forces behind social unrest: Evidence from the Covid-19 pandemic

**DOI:** 10.1371/journal.pone.0314165

**Published:** 2025-01-02

**Authors:** Mario Lackner, Uwe Sunde, Rudolf Winter-Ebmer

**Affiliations:** 1 JKU University of Linz, Linz, Austria; 2 University of Munich (LMU), München, Germany; 3 CEPR, London, United Kingdom; 4 IHS, Vienna, Austria; St John’s University, UNITED STATES OF AMERICA

## Abstract

The unprecedented consequences of the Covid-19 pandemic have raised concerns about the erosion of social cohesion and intensified social unrest, but evidence for such a link and the underlying channels is still lacking. We use a unique combination of nationally representative survey data, event data on social unrest, and data on Covid-19 fatalities and unemployment at a weekly resolution to investigate the forces behind social cohesion and unrest in the context of the strains on public health and the economy due to the pandemic in the USA. The results show that pandemic-related unemployment and Covid-19 fatalities intensified negative emotional stress and led to a deterioration of economic confidence among individuals. The prevalence of negative emotional stress, particularly in economically strained and politically polarized environments, was, in turn, associated with intensified social unrest as measured by political protests. No such link is found for economic perceptions.

## Introduction

The Covid-19 pandemic had unprecedented effects on public health and the economy. The pandemic caused more fatalities in the U.S. than all wars during the past century. The lockdown led to severe restrictions of civil liberties, brought the economy to a standstill, and resulted in the highest unemployment since the Great Depression. This has raised concerns of an intensification of social instability and social unrest. See, e.g., a recent op-ed in *Foreign Policy* by Elise Labott. Recent evidence indeed documented a deterioration of mental health, increasing depression rates, and an increase in household violence as consequences of the pandemic. Evidence whether this was also associated with a decline in social cohesion and an increase in social unrest is still lacking, however.

In this research, we provide novel evidence for a link between the Covid-19 pandemic and outbreaks of social unrest, as well as for the forces underlying this link. Conceptually, the analysis is motivated by the observation that the disruptions related to the Covid-19 pandemic pertain to several established determinants of social unrest that comprise personal grievances related to economic hardship, for instance due to job loss, to a negative economic outlook, or an aggravation of polarization. By intensifying existing socio-economic and political divides and leading to polarization in social norms regarding vaccination or more visible behaviors such as mask-wearing, the pandemic made some of these root causes of social unrest more prevalent. This gives rise to the conjecture of increasing social unrest. Our analysis is based on a unique combination of nationally representative weekly survey data collected during the pandemic with measures of the consequences of the pandemic such as daily data on Covid-19-related deaths at the state level, state-level statistics on unemployment, and geo-localized event-level data on protests and riots at a weekly resolution. The analysis covers the first Covid-19 phase (February through August, 2020) and concentrates on the role of political orientation and party opposition.

The results establish several novel findings regarding the link between negative emotional stress, economic perceptions related to the pandemic, and social unrest. First, we find evidence that individual job loss in the context of the Covid-19 pandemic led to an increased prevalence of negative emotional stress, anxiety, and aggression, as well as to increasingly negative perceptions of the state of the economy. These effects are largely unaffected by party affiliation or alignment of party preferences with the party of the state governor. Second, we document that similar effects emerge at the state-week-level when considering variation in deaths related to Covid-19 and in unemployment rates. In contrast to effects related to individual unemployment, here we also find systematic effect heterogeneity along party lines. Third, we document that an increase in negative emotional stress is associated with a significant increase in the incidence of social unrest at the state-week level. The fine grained data structure—combining data sources that have not been used previously—and the use of predetermined within-state and within-week variation documents a link between the Covid-19 pandemic and outbreaks of social unrest while ruling out endogeneity concerns. The estimates also show that negative emotional stress and negative perceptions about the economy are intensified by the severity of the crisis. Social unrest is related to greater negative emotional stress—but not to more negative perceptions about the economy. This effect is strongest in environments with a more pronounced increase in unemployment and Covid-19 fatalities, and in environments with a more polarized political climate, as reflected by narrow vote margins during the last gubernatorial election. The results are consistent with collective dissent as a consequence of a perceived decline of governmental legitimacy or diminishing confidence in the administrative capabilities to combat the pandemic, suggesting that in polarized contexts, in which individuals do not share common values, the enforcement of norms becomes more difficult and social cohesion is more fragile. Additional results document the robustness of these findings when accounting explicitly for Black Lives Matter protests.

In the remainder of the paper, we derive the hypotheses based on the conceptual background of our study, discuss the contribution in light of the existing literature, describe the data and methodology, present the results, and provide a brief discussion.

## Hypotheses

In this section, we derive several testable hypotheses from the existing literature to structure our analysis.

The Covid-19 pandemic triggered unprecedented public measures to contain the epidemic. In addition to the immediate stress related to potential infection, the economic impact and the widespread restrictions in private and public life might have contributed additional emotional stress due to social isolation [1]. School closures and the necessity of working from home presumably contributed to work-related emotional stress [2]. Other measures like wearing face-masks or contact tracing constituted serious inhibitions to personal freedom and might have affected emotional well-being [3, 4]. At the same time, employment shocks during the early phase of the Covid-19 pandemic are likely to have generated economic uncertainty related to job security and income prospects affecting individual economic outlooks (see, e.g. [5]). Taken together, these observations lead to our first hypothesis.

**Hypothesis 1** *Higher exposure to COVID-19 leads to more negative emotions and perceptions of the economy*.

Above and beyond individual-level experiences, economic fluctuations at the aggregate level might have similar effects. However, while individual experiences might reflect idiosyncratic factors, aggregate fluctuations are related to the broader political and economic environment and might thus be affected by political preferences and attitudes. For instance, it has been shown [6] that perceptions about the perceived Covid-19-related risks to health and economic conditions are influenced by partisanship, with individuals searching less for information about the virus and about welfare benefits and showing less reaction in terms of commuting behavior in areas with higher vote shares for Donald Trump. Related, [7] report interaction effects between partisan tendencies and attitudes regarding the policy responses to the pandemic. This gives rise to a second hypotheses:

**Hypothesis 2** *Negative emotions and perceptions of the economy are more polarized if triggered by aggregate events (like COVID-19 deaths or the unemployment rate) as compared to individual shocks*.

Grievances due to relative deprivation and economic uncertainty are among the main drivers of social unrest, particularly unrest related to issues of distributive and procedural social justice [8]. Apart from that, previous literature suggests that grievances alone do not lead to social unrest or protest unless enough resources for protest are available and individuals assume that their protest could be successful in the first place [9]. Evidence from social psychology suggests that emotions can act as accelerators or amplifiers of other unrest indicators [10]. Intergroup emotion theory stresses the importance of emotions, suggesting that the probability of protest and social unrest can increase when people experience emotions on behalf of their group. Anger is seen as the prototypical emotion in the context of unrest and group-based anger increases the willingness to participate in political action and protest [11].

The importance of efficacy for the probability of social unrest combines emotions with political polarization. If the political climate is more polarized and/or expected margins at the vote urn are smaller, protest and social unrest might be more efficient—and therefore, more protest is to be expected [8]. Moreover, the more people identify with a group, the more they are inclined to protest on behalf of that group [12]. In addition, a more strained economic situation can accelerate or amplify negative emotions and, thus, trigger social unrest. This leads to the following hypothesis:

**Hypothesis 3** *Emotional stress and negative perceptions of the economy are related to social unrest*.

Political partisanship may contribute to different perceptions of the same economic or political facts; even if they are confronted with the same facts, individuals may attribute these facts to different actors [13]—and thus different actions might arise.

From this follows

**Hypothesis 4** *The association between personal perceptions and social unrest is exacerbated in economically strained and politically polarized environments*.

In the analysis below, we explore the empirical relevance of each of these hypotheses using a unique combination of nationally representative survey data, event data on social unrest, and data on Covid-19 fatalities and unemployment at a weekly resolution.

## Literature review

Our findings contribute to the literature in several ways. The results document that the social and psychological consequences of the Covid-19 pandemic were associated with increased social unrest, in line with concerns mentioned early on during the pandemic (see, e.g., [14, 15]). Our results complement evidence of increased aggression levels during the pandemic-related lockdown [16, 17], increasing depression rates and a deterioration of mental health [18–20], and a significant loss in life satisfaction [21]. Moreover, they provide evidence for a link between the psychological fall-out of the pandemic and social unrest, complementing evidence for the effect of the pandemic on economic anxiety [22] and for increasing household violence during the Covid-19 pandemic [23, 24]. Our findings also contribute to the rather mixed evidence on the link between the pandemic and large-scale armed conflict [25–27] and to findings for increased social unrest related to Covid-19 in Africa, which was mainly due to the stringency of state reactions [28]. A survey on intimate partner violence, work-family conflict and East-Asian hate crimes related to Covid-19 is provided by [29].

The finding that social unrest can be traced to the disruptions caused by the Covid-19 pandemic supports the role of personal grievances as cause for collective dissent [30]. This finding is consistent with evidence for aggravated grievances as reflected by a negative perception of the economy [31, 32], a further widening of socio-ethnic inequalities [33], and health-related stress [34]. In light of evidence that the economic consequences of the pandemic are more widespread across age and regional groups than initial mortality impacts [35], our results potentially only capture the tip of the iceberg in terms of the social implications of the Covid-19 pandemic.

Moreover, our findings contribute to recent evidence that suggests that political polarization can lead to different interpretations of the same facts and policies related to the pandemic in various domains [13, 36–39]. Our findings document that comparable variation in unemployment or fatalities related to the pandemic entails very different consequences for social unrest depending on the political and economic context.

Our study also contributes to the literature on the link between epidemics and social unrest (see, e.g., [40], for a survey of historical cases). Existing evidence in this literature has mainly focused on epidemics and social violence in Africa (e.g., [34, 41]). Likewise, our findings complement macro-level evidence that suggests a link between epidemics and civil disorder world-wide [42]. Our empirical strategy contributes to this emerging literature by isolating the causal relation between Covid-19 and social unrest in the U.S. and by shedding light on the underlying psychological forces.

Finally, our findings contribute to a sizable and expanding literature on extreme contexts (for recent surveys of the literature on leadership and management, see, e.g., [43, 44]). In the context of the Covid-19 pandemic, this literature has increasingly considered strategies that corporations can use to manage extreme contexts and the associated social challenges and tensions (see, e.g. [45]). Moreover, several moderator variables such as gender or age have been isolated that influence the perception of extreme contexts and its implications for anxiety, alienation, and subjective job security (e.g. [46, 47]). By showing that the pandemic and party affiliation (respectively, party-related antagonism) interact in the context of social unrest, with significant consequences for the adherence to policies such as social distancing and the emergence of social norms related to transmission prevention, our estimates complement work on mass polarization of citizens’ positions in the past decades (see, e.g., [48–51]). There is also work on how selective media consumption changed perceptions and behavior in the pandemic [52].

## Data

### Data

The analysis combines survey data collected by the Gallup Covid-Survey, data on reported deaths related to the Covid-19 pandemic (https://github.com/datasets/covid-19), data on unemployment from the US Bureau of Labor statistics, and data on events of social unrest from the Global Database of Events, Language, and Tone (GDELT).

The Gallup COVID-19 survey was implemented as part of the Gallup panel and started on March 13 2020. The COVID-19 survey is a nationally representative panel, which was implemented random samples of persons aged 18 or older who were members of the Gallup panel, which at the time comprised approximately 80, 000 members. The survey collected 1, 000 interviews per day at the initial phase, later reducing the number of daily interviews to 500 per day (April 27, 2020), until August 17, 2020 when interviewing was reduced to monthly surveying. Interviews were conducted via the web and in English. Due to the randomized probability-based sampling, members were re-invited to interviews, but the panel structure of the data is highly unbalanced and time intervals are heterogenous. More than 48% of the respondents of our final sample were interviewed only one or two times. The average survey completion rate was 94%, the average response rate was 46% (ranging from 42% to 50.6%). Details of the methodology can be found in the Gallup Panel Covid-19 Codebook. As consequence of the infrequent and non-balanced panel structure of the data, we refrain from using the panel structure of the Gallup data by exploiting within-individual variation in our estimation model. In robustness analysis, we account for repeated observations by alternative clustering of the standard error on the individual level and document that our main results are confirmed.

The GDELT data is based on worldwide monitoring of print and online media to measure the extent of violent incidents and protest. See, https://www.gdeltproject.org/ for a detailed documentation. Overall, we consider the universe of media-documented events in the U.S. for the period February through August 2020 that was geolocated and assigned to a specific date by GDELT. In particular, our analysis of social unrest is based on protest events (event category 14) in the GDELT data. Despite potential deficiencies [53], these event data constitute the best available data source for the purpose of this analysis, which relies on high frequency (week-by-week-by-state) variation and an estimation strategy that flexibly accounts for potential confounders.

### Empirical methodology

The empirical analysis proceeds in three steps. In a first step, we document at the individual level that a job loss during the Covid-19 pandemic affected respondents’ overall sentiments. In the second step of the analysis, we use a similar approach to consider the socio-psychological effects of visible aggregate consequences of the Covid-19 pandemic on individual sentiments. The third step of the analysis investigates whether sentiments affect social unrest. In all three steps of the analysis, estimation relies on pre-determined variables of interest, which, on the second and third step of the analysis, are measured at the state-week level. This and the inclusion of a rich set of control variables that are predetermined at the time of measurement of the outcome, state and week fixed effects mitigates concerns of endogeneity or reverse causality and establishes identification of the coefficients of interest. In addition, robust inference is achieved by the explicit inclusion of potential correlation in the error at the state-week or state-month level, respectively. The next section provides a detailed account of the methods used in the three steps of the analysis and of the corresponding results.

## Results

### Individual pandemic-related unemployment shocks and sentiments

We start by presenting evidence on whether personal unemployment experience affects respondents’ sentiments at the individual level. The corresponding analysis is based on estimates of an empirical model

$$\begin{array}{c} SENT_{i,s,t}=\alpha +\beta UE_{i,s,t-1}+\xi^{\prime }X_{i,s,t}+\pi_{t}+\lambda_{s}+\varepsilon_{i,s,t}\;, \end{array}$$ (1)

with *SENT*_*i*,*s*,*t*_ denoting a binary dependent variable that measures the prevalence of negative sentiments, in terms of i) negative emotional stress or ii) a negative perception of the economy, for respondent *i* in week *t* in state *s*. Negative emotional stress is coded in terms of a binary variable that equals 1 if the principal-component score of negative emotional stress—based on survey responses to questions about anger, stress, worries, sadness, boredom, and loneliness—in the GALLUP-2020 (GALLUP 2020) questionnaire by respondent *i* in state *s* and calendar week *t* is in the top quartile, 0 otherwise. Detailed results for all components are presented in Table 1 in S1 File. Negative perception of the economy is a binary variable that equals 1 if the respondent reported to perceive the US economy to be in a depression, 0 otherwise (GALLUP 2020, item C13). *UE*_*i*,*s*,*t*−1_ is a pre-determined binary indicator whether the respondent reported a job loss during the previous week. ***X***_*i*,*s*,*t*_ is a vector of controls at the respondent-level, including household income, family status, children, educational attainment, ethnicity, age, a dummy indicating Republican affiliation, and a binary variable indicating political opposition to the state governor’s party affiliation, where opposition is defined as being affiliated with the opposite party, or, for independents, when the governor is Republican. The vector ***X***_*i*,*s*,*t*_ also includes controls for aggregate variation, including pandemic-related stay-at-home orders and social unrest in the week before the interview. Week (*π*_*t*_) and state (λ_*s*_) fixed-effects control for unobserved heterogeneity across time and regions. The error term *ε*_*i*,*s*,*t*_ allows for arbitrary correlation (clustering) at the state-week level.

**Table 1:** Descriptive statistics for main variables, by political party of state governour.

	*Pooled*		*Republican* ^a^		*non-Republican* ^b^		*diff*.^c^
	mean	(st.dev.)	mean	(st.dev.)	mean	(st.dev.)	
negative emotional stress (PCA)	0.23	(0.42)	0.22	(0.41)	0.25	(0.43)	0.03***
economic perception (*bad* = 1, *not*Â *bad* = 0)	0.26	(0.44)	0.24	(0.43)	0.28	(0.45)	0.04***
unemployed (*yes* = 1, *no* = 0)	0.08	(0.26)	0.07	(0.25)	0.08	(0.28)	0.02***
high deaths in *t*−1	0.65	(0.48)	0.60	(0.49)	0.69	(0.46)	0.09***
high unemployment *t*−1	0.63	(0.48)	0.62	(0.49)	0.65	(0.48)	0.03***
stay-at-home order in place	0.45	(0.50)	0.35	(0.48)	0.53	(0.50)	0.18***
social unrest in *t*−1	0.17	(0.34)	0.10	(0.17)	0.22	(0.41)	0.12***
in opposition (*yes* = 1, *no* = 0)	0.43	(0.50)	0.63	(0.48)	0.28	(0.45)	-0.34***
republican affiliation (*yes* = 1, *no* = 0)	0.32	(0.47)	0.37	(0.48)	0.28	(0.45)	-0.09***
children (*yes* = 1, *no* = 0)	0.21	(0.41)	0.21	(0.41)	0.21	(0.41)	0.00
married (*yes* = 1, *no* = 0)	0.69	(0.46)	0.71	(0.46)	0.68	(0.47)	-0.02***
non-white (*yes* = 1, *no* = 0)	0.12	(0.32)	0.11	(0.32)	0.12	(0.33)	0.01***
calender week of interview	21.16	(6.49)	21.15	(6.49)	21.16	(6.49)	0.01
*N*	92,567		40,061		52,506		

*Notes*:

^a^ States with a Republican governor in office.

^b^ States with a Democratic or independent governor in office.

^c^ Reported difference measures the difference in means for the non-Republican sample minus the Republican states;

*, ** and *** indicate statistical significance at the 10%, 5%, and 1% level for a t-test.

Table 1 presents descriptive statistics for the main variables used in the first part of our analysis. We present summary statistics for the overall sample we use, as well as for states governed by a republican or governor with other affiliation (Democrats or independent) during the study period. Based on our binary PCA measure, republican states exhibit slightly less negative emotions than democratic states. Respondents from non-Republican states have a slightly worse perception of the economy.

The experience of a job loss during the pandemic as reported in survey responses was associated with a significant intensification of negative emotional stress (Table 2 right Panel). A similar effect is found for responses regarding negative perceptions of the economy in terms of a depression (Table 2 right Panel). These findings are consistent with Hypothesis 1. In both cases, this estimate is affected only weakly by respondents’ political stance, in terms of considering an individual whose party affiliation does not align with the affiliation of the state governor (opposition) or party affiliation of the respondent (Republican or other).

**Table 2:** Association of individual unemployment experience with negative emotional stress and perception of the economy.

	(1)	(2)	(3)
*Panel A.* Negative emotions^a^			
unemployed in *t*−1^c^	0.121***	0.124***	0.114***
	(0.007)	(0.009)	(0.008)
unemployed in *t*−1 × opposition^d^		-0.008	
		(0.013)	
unemployed in *t*−1 × Republican^e^			0.026*
			(0.015)
opposition	0.004	0.004	0.003
	(0.003)	(0.003)	(0.003)
Republican	-0.086***	-0.086***	-0.087***
	(0.003)	(0.003)	(0.003)
stay-at-home	0.001	0.001	0.001
	(0.006)	(0.006)	(0.006)
social unrest in *t*−1	0.010	0.010	0.010*
	(0.006)	(0.006)	(0.006)
add. controls	*yes*	*yes*	*yes*
state, week FEs	*yes*	*yes*	*yes*
R^2^	0.044	0.044	0.044
mean dep. var.	0.226	0.226	0.226
*N*	70,789	70,789	70,789
*Panel B.* Negative perception of economy^b^			
unemployed in *t*−1^c^	0.071***	0.080***	0.074***
	(0.007)	(0.009)	(0.008)
unemployed in *t*−1 × opposition^d^		-0.021	
		(0.013)	
unemployed in *t*−1 × Republican^e^			-0.009
			(0.013)
opposition	-0.004	-0.002	-0.003
	(0.003)	(0.004)	(0.003)
Republican	-0.173***	-0.172***	-0.172***
	(0.003)	(0.003)	(0.004)
stay-at-home	0.005	0.005	0.005
	(0.007)	(0.007)	(0.007)
social unrest in *t*−1	-0.000	-0.000	-0.000
	(0.007)	(0.007)	(0.007)
add. controls	*yes*	*yes*	*yes*
state, week FEs	*yes*	*yes*	*yes*
R^2^	0.072	0.072	0.072
mean dep. var.	0.266	0.266	0.266
*N*	72,285	72,285	72,285

*Notes*: Results of linear probability models. All specifications include state and week fixed effects and the full set of controls. Standard errors, clustered on the state-week level, in parentheses,

*, ** and *** indicate statistical significance at the 10%, 5%, and 1% level.

^a^ binary dependent variable (mean 0.226).

^b^ binary dependent variable (mean 0.266).

^c^ Binary variable indicating individual unemployment status in *t*−1. Variable is equal to 1 if the respondent reported to be unemployed, 0 otherwise (GALLUP 2020, item E1_3).

^d^ Binary variable equal to 1 if the respondent reports party affiliation different than state governor’s party (for non-affiliates, opposition is coded when being resident in state with Republican governor).

^e^ Binary variable equal to 1 if the respondent reports affiliation with Republican party.

### Overall exposure to Covid-19 and sentiments

**Fig 1:**
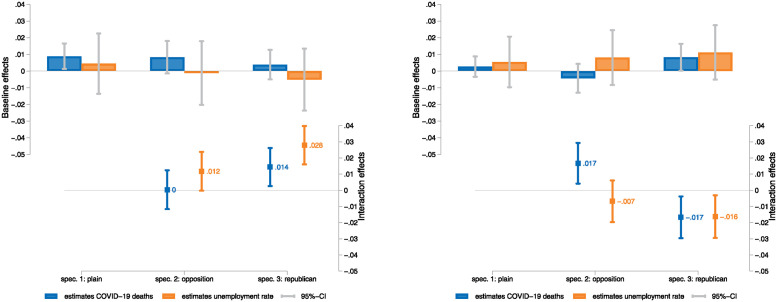
Association of Covid-19 deaths and unemployment rate with negative emotional stress and economic perception. *Notes:* Estimated coefficients and 95% confidence intervals. Left panel: Negative emotions (PCA). Right panel: Negative perception of economy. Estimation model for specification 1 (plain) as described in Eq 2. Specifications 2 and 3 include interactions of *COVID*_*s*,*t*−1_ and *UNEMP*_*s*,*t*−1_ with a binary variable indicating opposition (spec. 2) or a Republican affiliation indicator (spec. 3). Confidence intervals are based on standard errors that are clustered on the state-week level. Detailed estimates are reported in Table 2 of the S1 File.

In the second step of the analysis, we consider the effects of exposure to Covid-19, measured in terms of state-level fatalities and unemployment, on individual emotions and perceptions. The corresponding analysis is based on estimates of an empirical model

$$\begin{array}{c} SENT_{i,s,t}=\alpha +\beta COVID_{s,t-1}+\xi^{\prime }X_{i,s,t}+\pi_{t}+\lambda_{s}+\varepsilon_{i,s,t}\;, \end{array}$$ (2)

with *COVID*_*s*,*t*−1_ as a measure of exposure to the Covid-19 epidemic in terms of its consequences for public health and the economy. Exposure to Covid-19 is based on two proxies, state level unemployment and the rate of Covid-19-related fatalities, measured in state *s* for the preceding week *t*−1. Both proxies are highly visible, being permanently featured in the media, and pre-determined. We use Covid-19-related fatalities as a more visible and more severe indicator for Covid-19-related health effects as compared to the incidence of confirmed Covid-19 cases, which is measured with considerable error. In particular, we consider a measure of the consequences of the pandemic on public health in terms of a binary variable indicating a relatively high increase in the Covid-19 death rate (an increase in the death rate >9 per 1m from week *t*−2 to *t*−1, which corresponds to the median for the time period March through August), and the effect on the economy, as reflected by high unemployment (a monthly unemployment rate higher than 7%, which approximately corresponds to the sample median), at the state level. We update the monthly unemployment by using the previous month’s unemployment rate for the first 3 weeks of a month and the contemporary rate for weeks 4 and 5. The use of binary variables provides a useful source of variation without relying on a linear specification; the dynamics of the Covid-19 pandemic are clearly visible in the data (see Fig 1 in S1 File).

The results suggest that individual reactions are more polarized when considering exposure proxied by aggregate variables than when considering individual unemployment experience as above. In particular, increases in Covid-19 deaths at the state level are associated with a significant surge in negative emotional stress, whereas state-level unemployment rates do not affect emotions on average (Fig 1 (left), specification 1). At the same time, reactions to aggregate variation instead of individual experiences are more sensitive to partisanship. When considering heterogeneity by alignment of party affiliation with that of the state governor, there is no significant interaction with Covid-19 deaths on negative emotional stress, whereas increases in state-level unemployment intensify negative emotional stress among individuals in opposition to the state governor (spec. 2). It has been shown that confidence in political leaders can reduce the perceived riskiness of COVID-19 [54]. Party affiliation affects the emotional response to Covid-19 deaths and unemployment, with Republican-leaning respondents showing significantly stronger responses in terms of negative emotional stress to both types of events (spec. 3). This corresponds to results by [55] who find that after the unexpected election of Donald Trump Democrats became more pessimistic (and Republicans became more optimistic) with respect to the economy; at the same time, there were no systematic differences in expectations about their own personal situations. Note, there is also work on partisan beliefs about the economy [56].

The general association between Covid-19 casualties or unemployment and negative economic perceptions is weaker, but this conceals a considerable heterogeneity in the effects. The findings show that a high number of Covid-19-related deaths or high unemployment at the state level are not associated with a worse perception of the economy on average (Fig 1 (right), spec. 1). Yet, a high number of Covid-19 deaths is associated with a more pessimistic economic outlook of respondents who are in opposition with the governor; the corresponding effect for unemployment is insignificant (spec. 2). Both a high level of Covid-19-related deaths and unemployment at the state level, induce less of a negative perception of the economy among respondents who report an affiliation with the Republican party (spec. 3). This heterogeneity contrasts the heterogeneity found for negative emotional stress in Fig 1; the results suggest that the consequences of the pandemic in the economic and public health domain led to a greater prevalence of negative emotional stress and, to a lesser extent, of negative economic expectations, but the heterogeneity of these reactions reveals a widening of political divides and thus in the scope for social unrest. These results and those in Table 2 are in accordance with Hypothesis 2 that emotions and perceptions of the economy are more polarized with respect to political affiliation in more general as compared to personal settings.

Robustness checks using a binary dependent variable based on the PCA score of the second component yield no comparably systematic and significant results, suggesting that the psychological fall-out of the epidemic mainly pertains to anger-related negative emotional stress (angry, stressed, worried, sad) rather than introvertive negative emotions (boredom and loneliness). See Table 1 in the S1 File for the details of the factor analysis and Table 4 in S1 File for the corresponding estimation results for the second component. Unreported results also suggest no systematic heterogeneity in the effects for African American respondents.

### Sentiments and social unrest

The last step of the analysis investigates the link between sentiments and social unrest. In particular, we consider the association of protest events at the state-week-level with variation in negative emotional stress and perceptions expressed in the survey responses during the preceding week. The estimation analysis is based on weekly counts of social unrest as outcome variable and controls for stay-at-home orders that might have affected social unrest, as well as for nation-wide events unrelated to the Covid-19 pandemic, such as the Black Lives Matter movement [57], which gained momentum during the sample period. The corresponding analysis for this link between socio-psychological factors and unrest is based on the empirical model

$$\begin{array}{c} Unrest_{s,t}=\gamma_{0}+\gamma_{1}SENT_{s,t-1}+unemp_{s,t}+death\;rate_{s,t}+\varphi^{\prime }X_{s,t}+\rho_{t}+\lambda_{s}+\epsilon_{s,t}\;, \end{array}$$ (3)

with *Unrest*_*s*,*t*_ denoting the total number of registered events of social unrest in week *t* and state *s* in the GDELT data, *SENT*_*s*,*t*−1_ is a measure of sentiments in terms of negative emotional stress or economic perceptions as detailed in the figure notes. The controls for the unemployment rate (*unemp*_*s*,*t*_) and death rate (*death* *rate*_*s*,*t*_) in weeks *t* account for the direct effects of the pandemic dynamics. In addition, the vector ***X***_*s*,*t*_ includes state-week specific controls. In particular, to account for long-term trends in social unrest, we use 2018 and 2019 events in the same category, state and time period as additional controls. In addition, as the main variable of interest is measured in *t*−1, we include the recorded number of protests in week *t*−2 as additional control variable to account for other unobserved confounders or mean reversion. Week (*ρ*_*t*_) and state (λ_*s*_) fixed-effects control for the overall dynamics and systematic heterogeneity reflected in social unrest. In sum, this empirical specification accounts for the direct effects of Covid-19 on social unrest and, in combination with the high temporal resolution of the data, it allows identifying the effect of socio-psychological factors captured by the sentiments variable. The error term *ϵ*_*s*,*m*,*t*_ allows for arbitrary correlation (clustering) at the state-month level.

**Fig 2:**
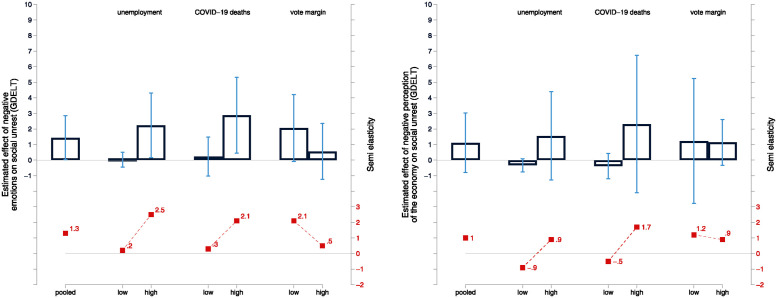
Negative emotions, economic perceptions, and social unrest. *Notes:* Estimated coefficients and 95% confidence intervals. Coefficient estimates are derived from weighted least squares regressions of Eq 3, weighted by the standard deviation of the dependent variable on the state-week level. Left panel: Association of negative emotional stress (PCA) with social unrest. Right panel: Association of economic perception with social unrest. Only state-week combinations with more than 10 valid survey responses for both types of sentiments are considered. Washington D.C. is excluded. Semi-elasticity (calculated using the unweighted mean of the respective sample as the base) multiplied by 100 gives the percentage change in the specific rate (ratio) due to a one percentage point increase in the mean of the PCA and economic perception variables. Standard errors are clustered at the state-month level. Detailed estimates are reported in Table 3 of the S1 File.

The estimates document that, on average, the prevalence of negative emotional stress in a particular week and state exhibits a significant positive association with the occurrence of events of social unrest (Fig 2 (right panel), pooled). With an increase in social unrest, measured by GDELT protest events, of 1.3 percent in response to an increase in the prevalence of negative emotional stress in the population by 1 percentage point, this effect is sizable. This result provides support for Hypothesis 3.

It is remarkable that this effect does not arise in environments with a relatively low level of Covid-19-related fatalities or in politically polarized environments with low (i.e., close) vote margins. On the other hand, the association between negative emotional stress and social unrest is systematically stronger in states and weeks in which unemployment rates exceed 7%, which approximately corresponds to the sample median, while there is no significant link between negative emotional stress and social unrest in a low unemployment context. A similar heterogeneity emerges for differences in (cumulative) Covid-19 deaths. In states and weeks in which the growth in the death rate was larger than 9 per 1m, which is about the median for the time period March through August, the link between average prevalence of negative emotional stress and social unrest is positive and significant, in contrast to environments with a low number of cumulated deaths. The effect heterogeneity in the link between negative emotional stress and social unrest also pertains to political polarization in terms of small vote margins. In particular, in states with high polarization as reflected by a low vote margin between Democrats and Republicans during the most recent gubernatorial election, a higher average prevalence of negative e+motional stress is associated with a significantly higher incidence of social unrest, whereas this link is insignificant in states with a high vote margin. The finding of a larger reaction of social unrest with respect to the prevalence of emotional stress for all three proxies of a politically polarized environment and thus amplifying thus personal stress or grievances, provides empirical support for Hypothesis 4.

When considering economic perceptions, the results reveal no significant link between average economic perceptions and social unrest (Fig 2 (right panel), pooled). While qualitatively similar, the patterns of heterogeneity in the association between economic perceptions and social unrest is quantitatively weaker and not statistically significant. This evidence suggests that social unrest is more closely associated with psychological factors related to negative emotional stress, than with economic perceptions and expectations. While the baseline analysis is based on a binary variable indicating negative emotional stress, we also estimated a model using the state-week average of the continuous measure for negative emotions (i.e., the PCA score) as the explanatory variable to explore the robustness of our findings. The corresponding estimates confirm all findings presented in Fig 2, see Table 5 in the S1 File.

In sum, the evidence presented here suggests that the psychological fall-out of the Covid-19 pandemic in terms of an increased prevalence of negative emotional stress exhibits a significant association with increased social unrest. The corresponding association between perceptions of the state of the economy and social unrest is found to be insignificant.

### Robustness

The effect heterogeneity indicates that political polarization acts as catalyst that contributes to increased economic and political tensions: the association between negative emotional stress and social unrest is primarily found in the context of high unemployment or high Covid-19 fatalities or in situations of high political polarization at the state level.

The finding of effect heterogeneity does not hinge on the particular specification of the thresholds for unemployment and Covid-19 fatalities used to partition the sample. In a series of sensitivity checks, we define different cut-off values for the unemployment rates (from 6 to 8 percent) and Covid-19 death rates (from 5 to 13 per 1m). The estimates confirm our main results and are presented in Figs 2 and 3 in S1 File. Likewise, the findings are not sensitive to dropping particular states (see Figs 4 and 5 in S1 File).

The simultaneous occurrences of social unrest related to police violence and the Black Lives Matter (BLM) movement has been viewed as part of a broader process of deepening racial and socio-economic divides that has been accelerated and exposed by the Covid-19 pandemic [15]. The estimation results are unlikely to be affected by outbreaks of violence at selective dates, such as in the aftermath of the killing of George Floyd, due to the inclusion of state and week fixed effects that account for the spatial and temporal concentration of such events. In addition, seasonal trends in social unrest are accounted for by controlling for events during the same periods in years 2018 and 2019.

Nevertheless, we conducted several robustness checks to explore whether the main finding in fact masks the rise in social unrest related to the Black Lives Matter movement. Estimation results of extended specifications deliver no evidence for systematic heterogeneity in the effects of negative emotional stress on social violence across states with low or high population shares of African Americans; if anything, the results are slightly weaker in states with higher population shares, pointing in the opposite direction (see Table 6 in S1 File). Additional results document that the main findings are robust when using a more extensive specification of the empirical model (see Tables 7 and 8 in S1 File). Table 7 in S1 File shows that negative emotional stress is significantly correlated with social unrest whether or not fixed effects for states or weeks (Cols. (1)—(3)) are introduced. To control for the prevalence of BLM protests, in Cols (4) and (5) we include specific state-level control variables, like population, density, the share of black population, crime and imprison rates as well as the poverty rate. The results also remain unchanged when including a dummy for states and weeks when the Black Lives Matter protests were most severe (Col. (6)). The same holds when excluding entire state-weeks (Col. (7)) or states (Col (8)) in which BLM protests have been severe. All estimates presented in Table 7 in S1 File suggest that our finding of a positive association between emotional distress and social unrest is not due to BLM protests coinciding with the end of the first Covid-19 phase.

## Discussion

This paper documents several findings based on a unique combination of nationally representative survey data, event data on social unrest, and data on Covid-19 fatalities and unemployment at a weekly resolution. First, higher exposure to COVID-19 leads to more negative emotions and perceptions of the economy. In line with recent evidence that employment shocks affect individual economic outlooks (see, e.g. [5]), we find that individuals experienced increasing economic uncertainty related to job security and income prospects, which also led to increased negative emotions.

Second, negative emotions and perceptions of the economy are more polarized in response to aggregate events (like COVID-19 deaths or the unemployment rate) than in response to individual shocks. While individual experiences might reflect idiosyncratic factors, aggregate fluctuations are related to the broader political and economic environment and might thus be affected by political preferences and attitudes. This is consistent with recent findings that perceptions about the perceived Covid-19-related risks to health and economic conditions are influenced by partisanship, with individuals searching less for information about the virus and about welfare benefits and showing less reaction in terms of commuting behavior in areas with higher vote shares for Donald Trump [6]. Related a study reports [7] interaction effects between partisan tendencies and attitudes regarding the policy responses to the pandemic, with the widespread restrictions in private and public contributing additional emotional stress due to social isolation [1], school closures and the necessity of working from home [2]. Other measures like wearing face-masks or contact tracing constituted serious inhibitions to personal freedom and might have affected emotional well-being, while compliance was heavily influenced by political partisanship [3, 4, 58].

Third, emotional stress and negative perceptions of the economy are related to an erosion of social norms and cohesion and a surge of social unrest. Evidence from social psychology suggests that emotions can act as accelerators or amplifiers of other unrest indicators [10]. At the same time, grievances due to relative deprivation and economic uncertainty are among the main drivers of social unrest, particularly unrest related to issues of distributive and procedural social justice [8]. While the evidence shown here does not necessarily reflect causal effects, the fine grained data structure and the use of predetermined within-state and within-week variation provides novel insights into the link between the Covid-19 pandemic and outbreaks of social unrest that complement recent findings regarding the role of Covid-19 for affective polarization (see, e.g. [7]).

Fourth, the association between personal perceptions and social unrest is exacerbated in economically strained and politically polarized environments. The importance of emotions for social cohesion and social unrest, particularly when people experience emotions on behalf of their group, has been documented before, with (group-based) anger being the prototypical emotion in the context of unrest determining the willingness to participate in political action and protest [11]. At the same time, if the political climate is more polarized, as reflected by closer vote margins, protest and social unrest is more likely due to higher group identification and greater perceived effectiveness of protests [8, 12]. Thus, the combination of increasing emotional stress related to economic conditions and a more polarized reaction to the policy responses to Covid-19 ultimately led to greater social unrest.

In sum, the evidence presented here suggests that the psychological fall-out of the Covid-19 pandemic—in terms of an increased prevalence of negative emotional stress—exhibits a significant association with increased social unrest that is amplified in politically polarized environments, whereas perceptions of the state of the economy do not seem to correlate with social unrest. Our results thus suggests an important interaction between psychological factors and social cohesion: The acceptance and effectiveness of policy responses is closely linked to emotional responses, which are determined by aggregate fluctuations and by the prevalence of group-level polarization in terms of political partisanship. An implication of our findings is that policies regarding the prevention of, and reaction to, social unrest need to consider that in polarized contexts, in which individuals do not share common values or perceptions, the enforcement of norms becomes more difficult and social cohesion is more fragile. Moreover, individual moods react in a more partisan way to general economic (or societal) conditions and less so to personal problems. This has consequences for information campaigns related to policies as well as measures for conflict prevention.

There are some limitations to this study. First, the analysis concentrates on a single, major disruptive event: the Covid-19 pandemic. Reactions to other—less extreme—contexts may be less dramatic and our results may, thus, not be fully generalizable. The analysis also focuses on the United States, a country that has experienced significant social polarization in recent years, which again might affect the generalizability of the results to less polarized countries. Further, we are using a combination of survey data from Gallup with real data from social unrest. Despite the advantages of this approach, the replicability of the results in other contexts might depend on the elicitation of survey data with different sampling schemes and less renowned survey companies. Finally, more work is needed for a better understanding of the forces underlying the interactions between aggregate fluctuations, group-level polarization, and social conflict, and the adequate policy responses to prevent or attenuate the related outbreaks of violence.

## Supporting information

S1 File Appendix: Additional Figures and Tables. (PDF)
