# Supplementary material for: The forces behind social unrest: Evidence from the Covid-19 pandemic
Source: PLoS One. 2025 Jan 2;20(1):e0314165. doi: 10.1371/journal.pone.0314165 (PMC11695001; doi:10.1371/journal.pone.0314165)
Supplement: S1 File — (PDF) [file pone.0314165.s001.pdf]

Additional Figures and Tables

Fig 1. Dynamics of indicators of high COVID-19-related deaths and high unemployment

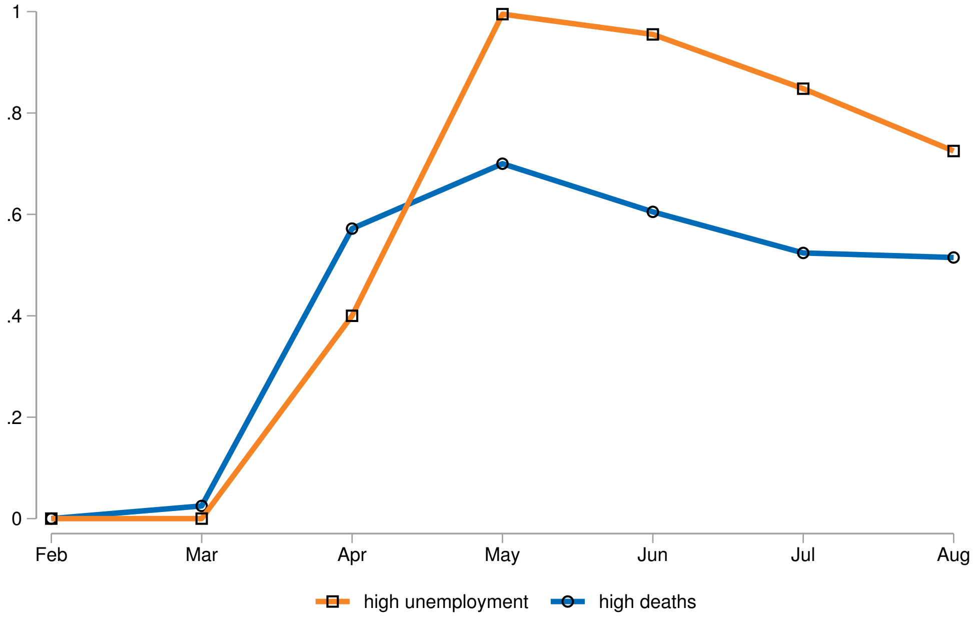

Notes: The graph illustrates the mean of the binary variable indicating high COVID-19 deaths, as well as the unemployment rate for months February through August 2021.

Table 1. Principle component analysis

Variable	**Comp. 1**	**Comp. 2**	**Comp. 3**	**Comp. 4**	**Comp. 5**	**Comp. 6**
angry	0.3882	−0.2545	0.6857	0.3972	0.3927	−0.0491
stressed	0.4562	−0.2694	−0.5176	0.0476	0.3046	0.5969
worried	0.4924	−0.2202	−0.3767	0.0835	−0.1478	−0.7337
sad	0.4765	−0.0351	0.3141	−0.2894	−0.7147	0.2801
bored	0.2184	0.7254	−0.1161	0.6167	−0.1555	0.0900
lonely	0.3520	0.5354	0.0885	−0.6074	0.4430	−0.1283
Eigenvalues	2.3988	1.1255	0.7842	0.6990	0.5553	0.4373

Notes: $N = 101,302$, all variables are binary and taken from the GALLUP (2020) survey.

Table 2. Association of unemployment and COVID-19 shocks on negative emotional stress and perception of the economy - full estimation results for Figure 1 in manuscript

	negative emotional stress[a]			*economic perception*[b]		
	(1)	(2)	(3)	(4)	(5)	(6)
high deaths in $t-1$[c]	0.009**	0.008*	0.004	0.003	−0.004	0.008**
	(0.004)	(0.005)	(0.005)	(0.003)	(0.004)	(0.004)
high unemp. in $t-1$[d]	0.004	−0.001	−0.005	0.005	0.008	0.011
	(0.009)	(0.010)	(0.009)	(0.008)	(0.008)	(0.008)
high deaths in $t-1$		0.000			0.017***	
× opposition		(0.006)			(0.006)	
high unemp. in $t-1$		0.012*			−0.007	
× opposition		(0.006)			(0.007)	
high deaths in $t-1$			0.014**			−0.017**
× Republican			(0.006)			(0.007)
high unemp. in $t-1$			0.028***			−0.016**
× Republican			(0.006)			(0.007)
opposition	0.003	−0.004	0.002	−0.006*	−0.012**	−0.005
	(0.003)	(0.006)	(0.003)	(0.003)	(0.006)	(0.003)
Republican	−0.098***	−0.098***	−0.125***	−0.169***	−0.170***	−0.148***
	(0.003)	(0.003)	(0.005)	(0.003)	(0.003)	(0.006)
stay-at-home[f]	−0.001	−0.001	−0.001	0.003	0.003	0.004
	(0.005)	(0.005)	(0.005)	(0.005)	(0.005)	(0.005)
social unrest in $t-1$[g]	0.008	0.009	0.009	0.002	0.002	0.002
	(0.006)	(0.006)	(0.006)	(0.007)	(0.007)	(0.006)
social unrest 2018/2019	*yes*	*yes*	*yes*	*yes*	*yes*	*yes*
add. controls	*yes*	*yes*	*yes*	*yes*	*yes*	*yes*
week FEs	*yes*	*yes*	*yes*	*yes*	*yes*	*yes*
state FEs	*yes*	*yes*	*yes*	*yes*	*yes*	*yes*
R^2	0.041	0.041	0.041	0.065	0.065	0.065
Mean dep. var.	0.234	0.234	0.234	0.263	0.263	0.263
N		90,396			92,277	

Notes: Robust standard errors, clustered on the state-week level, are presented in parentheses, stars indicate significance: *, ** and *** indicate statistical significance at the 10-percent level, 5-percent level, and 1-percent level. Estimation model is as described in equation 1. All result correspond to those illustrated in Figure 1.
[a] The dependent variable is equal to 1 if the PCA score of negative emotional stress according to the GALLUP-2020 survey is in the top quartile, 0 if in the lower 3 quartiles. [b] The dependent variable is equal to 1 the GALLUP-2020 survey respondent reported to perceive the US economy to be in a recession, 0 otherwise. [c] Binary variable equal to 1 if the change in the observed cumulative COVID-19 related death rate from $t-2$ to $t-1$ (deaths per 1m state population) is above 9, 0 otherwise. [d] Binary variable equal to 1 if the observed unemployment rate of a particular week in a particular state is above the median nation-wirde unemployment rate (~ 7) during weeks 4–35, 0 otherwise. [e] Binary variable indicating individual unemployment status. Variable is equal to 1 if the respondent reported to be unemployed, 0 otherwise. [f] Binary variable equal to 1 if observed week was affected by state-wide stay-at-home order in $t-1$, 0 otherwise. [g] social unrest as recorded by GDELT category 14, rescaled by 1000. States-week combinations with only selected counties affected by stay-at-home order are coded as 0. Additional controls on respondent-level include household income, family status, children, educational attainment, ethnicity, and age.

Table 3. Association of negative emotion and negative perception of the economy and social unrest - full estimation output for Figure 2 in manuscript

		unemployment		*COVID-19 deaths*		*vote margin*	
	(1)	(2)	(3)	(4)	(5)	(6)	(7)
	pooled	low	high	low	high	low	high
Panel A. Negative emotions and political protest[a]							
avg. negative emotions (PCA)[a]	1.443**	0.033	2.241**	0.228	2.886**	2.061*	0.561
	(0.716)	(0.242)	(1.052)	(0.634)	(1.235)	(1.086)	(0.906)
semi elasticity[c]	[1.3]	[0.2]	[2.5]	[0.3]	[2.1]	[2.1]	[0.5]
unemployment rate $t-1$	−4.228	0.216	−10.593*	−0.123	−4.429	−4.842	−4.512
	(3.540)	(0.754)	(5.734)	(3.196)	(3.855)	(4.368)	(6.785)
death rate $t-1$	0.057	−0.009	0.040	−0.036	0.128	−0.043	0.093
	(0.054)	(0.022)	(0.179)	(0.023)	(0.100)	(0.047)	(0.078)
social unrest $t-2$	0.151**	0.166	0.083	0.293***	0.084	0.114	0.181**
	(0.060)	(0.141)	(0.070)	(0.107)	(0.078)	(0.078)	(0.070)
social unrest 2018 & 2019	*yes*	*yes*	*yes*	*yes*	*yes*	*yes*	*yes*
week FEs	*yes*	*yes*	*yes*	*yes*	*yes*	*yes*	*yes*
state FEs	*yes*	*yes*	*yes*	*yes*	*yes*	*yes*	*yes*
R^2	0.497	0.829	0.507	0.531	0.553	0.393	0.581
Panel B. Negative perception of the economy and political protest[b]							
avg. economic perception (BAD ECON)[b]	1.114	−0.334	1.560	−0.385	2.317	1.233	1.140
	(0.972)	(0.214)	(1.438)	(0.414)	(2.236)	(2.029)	(0.744)
semi elasticity[c]	[1.0]	[−0.9]	[0.9]	[−0.5]	[1.7]	[1.2]	[0.9]
unemployment rate $t-1$	−2.397	0.099	−9.751*	2.990	−3.762	−5.501	0.057
	(3.516)	(0.673)	(5.624)	(3.236)	(3.815)	(5.756)	(4.769)
death rate $t-1$	0.062	0.000	0.009	−0.036	0.106	0.002	0.084
	(0.049)	(0.023)	(0.154)	(0.027)	(0.075)	(0.067)	(0.068)
social unrest $t-2$	0.168***	0.210	0.072	0.345***	0.101	0.135*	0.206***
	(0.060)	(0.145)	(0.073)	(0.114)	(0.080)	(0.080)	(0.063)
social unrest 2018 & 2019	*yes*	*yes*	*yes*	*yes*	*yes*	*yes*	*yes*
week FEs	*yes*	*yes*	*yes*	*yes*	*yes*	*yes*	*yes*
state FEs	*yes*	*yes*	*yes*	*yes*	*yes*	*yes*	*yes*
R^2	0.454	0.840	0.477	0.520	0.514	0.359	0.565
Observations	1,052	343	709	440	612	539	513

Notes: Estimation results corresponding to Figure 2 in the manuscript. Heteroskedasticity robust standard errors are presented in parentheses, stars indicate significance: *, ** and *** indicate statistical significance at the 10-percent level, 5-percent level, and 1-percent level. All estimates are derived from weighted least squares regression, weighting by the standard deviation of the dependent variable at the state-week level. Estimation model is as described in equation 2. All result correspond to those illustrated in Figure 2. The same definitions of high/low Covid-19 deaths and unemployment rate are used as in Table 2. The vote margin split is defined along the across-state median of the margin of the most recent gubernatorial elections. [a] Mean negative emotional stress reported on state-week level in percent. It measures the percentage of respondents in GALLUP-20 who registered a PCA score in the upper quartile. [b] The economic perception variable measures the percentage of GALLUP-2020 survey respondents who reported to perceive the US economy to be in a recession. [c] Semi-elasticity (calculated using the unweighted mean of the respective sample as the base) multiplied by 100 gives the percentage change in the specific rate (ratio) due to an one percentage point increase in the mean of the PCA and economic perception variables.

Table 4. Association of unemployment and COVID-19 shocks on negative emotional stress - second component

	second component[a]		
	(1)	(2)	(3)
high deaths in $t{-}1^c$	0.002	−0.002	0.001
	(0.004)	(0.005)	(0.005)
high unemp. in $t{-}1^d$	0.000	−0.001	0.006
	(0.010)	(0.011)	(0.010)
high deaths in $t{-}1$		0.009	
× opposition		(0.008)	
high unemp. in $t{-}1$		0.001	
× opposition		(0.008)	
high deaths in $t{-}1$			0.005
× Republican			(0.008)
high unemp. in $t{-}1$			−0.020**
× Republican			(0.008)
opposition	0.004	−0.002	0.004
	(0.004)	(0.006)	(0.003)
Republican	0.007**	0.007*	0.017**
	(0.004)	(0.004)	(0.006)
stay-at-home[f]	0.011*	0.011*	0.011*
	(0.006)	(0.006)	(0.006)
social unrest in $t{-}1$	0.008	0.008	0.008
	(0.006)	(0.006)	(0.006)
social unrest 2018/2019	*yes*	*yes*	*yes*
add. controls	*yes*	*yes*	*yes*
week FEs	*yes*	*yes*	*yes*
state FEs	*yes*	*yes*	*yes*
R^2	0.031	0.031	0.031
Mean dep. var.	0.234	0.234	0.234
N		90,396	

Notes: Robust standard errors, clustered on the state-week level, are presented in parentheses, stars indicate significance: *, ** and *** indicate statistical significance at the 10-percent level, 5-percent level, and 1-percent level. Estimation model is as described in equation 1. All result correspond to those illustrated in Figure 1.

[a] The dependent variable is equal to 1 if the PCA score of the second component of negative emotional stress according to the GALLUP-2020 survey is in the top quartile, 0 if in the lower 3 quartiles. [c] Binary variable equal to 1 if the observed cumulative COVID-19 related death rate (deaths per 1m state population) is above the median ($\sim$ 70), 0 otherwise. [d] Binary variable equal to 1 if the observed unemployment rate of a particular week in a particular state is above the median nation-wirde unemployment rate ($\sim$ 7.5) during weeks 4–35, 0 otherwise. [e] Binary variable indicating individual unemployment status. Variable is equal to 1 if the respondent reported to be unemployed, 0 otherwise. [f] Binary variable equal to 1 if observed week was affected by state-wide stay-at-home order, 0 otherwise. [g] political protest as recorded by GDELT category 14, rescaled by 1000. States-week combinations with only selected counties affected by stay-at-home order are coded as 0. Additional controls on respondent-level include household income, family status, children, educational attainment, ethnicity, and age.

Table 5. Association of negative emotion and social unrest - continuous measure of negative emotional stress (PCA score)

		unemployment		*COVID-19 deaths*		*vote margin*	
	(1)	(2)	(3)	(4)	(5)	(6)	(7)
	pooled	low	high	low	high	low	high
avg. negative emotions (PCA)[a]	70.960**	−2.474	94.313**	18.807	115.409**	87.299*	41.772
	(30.231)	(5.741)	(43.363)	(18.504)	(52.487)	(50.161)	(31.408)
unemployment rate $t-1$	−4.296	0.218	−9.845*	−0.095	−4.503	−4.865	−4.287
	(3.547)	(0.725)	(5.548)	(3.123)	(3.779)	(4.539)	(6.578)
death rate $t-1$	0.061	−0.007	0.038	−0.037	0.137	−0.029	0.096
	(0.054)	(0.022)	(0.171)	(0.023)	(0.099)	(0.052)	(0.078)
social unrest $t-2$	0.143**	0.158	0.080	0.286***	0.079	0.109	0.175**
	(0.060)	(0.142)	(0.069)	(0.107)	(0.078)	(0.080)	(0.070)
social unrest 2018 & 2019	*yes*	*yes*	*yes*	*yes*	*yes*	*yes*	*yes*
week FEs	*yes*	*yes*	*yes*	*yes*	*yes*	*yes*	*yes*
state FEs	*yes*	*yes*	*yes*	*yes*	*yes*	*yes*	*yes*
R^2	0.494	0.823	0.505	0.530	0.553	0.393	0.581
Observations	1,052	343	709	440	612	539	513

Notes: Estimation results corresponding to Figure 2 in the manuscript. Heteroskedasticity robust standard errors are presented in parentheses, stars indicate significance: *, ** and *** indicate statistical significance at the 10-percent level, 5-percent level, and 1-percent level. All estimates are derived from weighted least squares regression, weighting by the standard deviation of the dependent variable at the state-week level. Estimation model is as described in equation 2. All result correspond to those illustrated in Figure 2. The same definitions of high/low Covid-19 deaths and unemployment rate are used as in Table 2. The vote margin split is defined along the across-state median of the margin of the most recent gubernatorial elections. [a] Mean score (by state-week) core calculated from component 1 of the principal component analysis for all 6 GALLUP-2020 items indicating negative emotional stress, mean −0.090 (st.dev. 0.306).

Table 6. Association of negative emotion and social unrest - interaction Afro-American population

		unemployment		*COVID-19 deaths*		*vote margin*	
	(1)	(2)	(3)	(4)	(5)	(6)	(7)
	pooled	low	high	low	high	low	high
avg. negative emotions (PCA)[a]	1.791**	−0.223	2.969**	0.115	3.606**	2.169*	1.176
	(0.870)	(0.236)	(1.344)	(0.669)	(1.671)	(1.249)	(1.175)
avg. negative emotions	−1.387	1.481	−2.563	0.773	−2.086	−0.618	−1.791
× high Afro American pop.[b]	(1.508)	(0.978)	(2.129)	(1.426)	(2.372)	(1.745)	(2.350)
additional controls	*yes*	*yes*	*yes*	*yes*	*yes*	*yes*	*yes*
week FEs	*yes*	*yes*	*yes*	*yes*	*yes*	*yes*	*yes*
state FEs	*yes*	*yes*	*yes*	*yes*	*yes*	*yes*	*yes*
R^2	0.497	0.831	0.508	0.531	0.553	0.393	0.582
Observations	1,052	343	709	440	612	539	513

Notes: Estimation results corresponding to Figure 2 in the manuscript. Heteroskedasticity robust standard errors are presented in parentheses, stars indicate significance: *, ** and *** indicate statistical significance at the 10-percent level, 5-percent level, and 1-percent level. All estimates are derived from weighted least squares regression, weighting by the standard deviation of the dependent variable at the state-week level. Estimation model is as described in equation 2. All result correspond to those illustrated in Figure 2. The same definitions of high/low Covid-19 deaths and unemployment rate are used as in Table 2. The vote margin split is defined along the across-state median of the margin of the most recent gubernatorial elections. [a] Mean score (by state-week) core calculated from component 1 of the principal component analysis for all 6 GALLUP-2020 items indicating negative emotional stress, mean −0.090 (st.dev. 0.306). [b] Interaction of PCA variable with a binary variable indicating a high share of Afro American population in the observed state. Threshold value is 14%, the mean of the Afro American population (according to the 2019 census) is 13.4%.

Table 7. Association of negative emotional stress (PCA) and social unrest – alternative specifications

	(1)	(2)	(3)	(4)	(5)	(6)	(7)	(8)
PCA	1.796**	1.443**	1.455*	2.149***	2.201***	1.972***	1.465**	2.028**
	(0.739)	(0.716)	(0.826)	(0.780)	(0.778)	(0.757)	(0.647)	(0.876)
social unrest $t{-}2$	0.246***	0.151**	0.150**	0.210***	0.205***	0.189***	0.268***	0.225***
	(0.047)	(0.060)	(0.061)	(0.048)	(0.049)	(0.052)	(0.049)	(0.073)
unemp. rate $t{-}1$		−4.228	−4.296	−4.033	−3.102	−3.153	−0.340	−2.385
		(3.540)	(3.612)	(3.193)	(2.940)	(2.658)	(1.278)	(2.282)
death. rate $t{-}1$		0.057	0.065	0.061	0.060	0.058	0.003	0.047
		(0.054)	(0.057)	(0.041)	(0.041)	(0.038)	(0.013)	(0.046)
state pop. (mio.)				10.272***	10.991***	9.868***	7.949**	10.604**
				(2.971)	(3.106)	(3.099)	(3.418)	(5.025)
pop. density				−0.083**	−0.119**	−0.077***	−0.039***	−0.073**
				(0.033)	(0.046)	(0.029)	(0.015)	(0.035)
black share				−95.150	1.944	−98.140	−53.985	−113.856
				(65.423)	(73.142)	(62.683)	(48.254)	(72.239)
Republican				−42.472**	−38.633**	−45.492***	−30.291***	−15.798
				(17.044)	(15.443)	(17.101)	(10.745)	(11.560)
vote margin				0.707	0.691	0.659	0.585	0.404
				(0.535)	(0.524)	(0.561)	(0.514)	(0.673)
stay-at-home				−12.656	−13.951	−0.891	20.084	4.176
				(22.378)	(22.978)	(18.118)	(13.987)	(38.839)
crime rate					−0.051			
					(0.032)			
imprison rate					−0.061			
					(0.063)			
poverty rate					−1.000			
					(2.632)			
strong blm						192.322*		
						(113.303)		
week FEs	yes	yes	yes	yes	yes	yes	yes	yes
state FEs	no	yes	yes	no	no	no	no	no
week*black-share FEs	no	no	yes	no	no	no	no	no
N	1,052	1,052	1,052	1,052	1,052	1,052	993	678
R^2	0.423	0.497	0.498	0.458	0.460	0.476	0.490	0.488

Notes: Robust standard errors, are presented in parentheses, stars indicate significance: *, ** and *** indicate statistical significance at the 10-percent level, 5-percent level, and 1-percent level. Specification (2) is our main specification as presented in equation 2.
Additional state-level control variables in specification (5) are derived from d the FBI Uniform Crime Reporting Program. Data are available at `https://eu.usatoday.com/story/money/2020/01/13/most-dangerous-states-in-america-violent-crime-murder-rate/40968963/`
The variable *strong BLM* is equal to 1 if the increase in registered political protest at the beginning of the Black Lives Matter protest was larger than 11, 0 otherwise.
Specification (7) is estimated using model (4) without state-weeks that are strongly affected by the Black Lives Matter protests.
Specification (8) is estimated using model (4) without states that are above-median affected by the Black Lives Matter protests.

Table 8. Association of negative perception of the economy and social unrest – alternative specifications

	(1)	(2)	(3)	(4)	(5)	(6)	(7)	(8)
BAD ECON	1.121	1.040	1.134	1.334	1.360	1.226	0.153	0.754
	(0.758)	(0.935)	(1.015)	(0.878)	(0.827)	(0.821)	(0.517)	(0.826)
social unrest $t-2$	0.246***	0.152**	0.151**	0.211***	0.206***	0.190***	0.267***	0.224***
	(0.046)	(0.060)	(0.060)	(0.047)	(0.048)	(0.052)	(0.050)	(0.074)
unemp. rate $t-1$		−4.089	−4.103	−4.066	−3.110	−3.179	−0.195	−2.253
		(3.456)	(3.513)	(3.266)	(2.987)	(2.720)	(1.250)	(2.304)
death. rate $t-1$		0.053	0.060	0.062	0.061	0.059	0.002	0.046
		(0.053)	(0.056)	(0.042)	(0.042)	(0.039)	(0.013)	(0.047)
state pop. (mio.)				10.276***	10.945***	9.870***	7.844**	10.634**
				(2.985)	(3.113)	(3.113)	(3.437)	(5.070)
pop. density				−0.080**	−0.113**	−0.074**	−0.032**	−0.067*
				(0.034)	(0.046)	(0.030)	(0.014)	(0.035)
black share				−88.050	0.341	−91.608	−61.509	−111.747
				(63.256)	(72.667)	(60.806)	(48.638)	(70.421)
Republican				−41.135**	−37.695**	−44.274***	−31.928***	−15.402
				(16.832)	(15.719)	(16.750)	(11.313)	(12.534)
vote margin				0.622	0.630	0.581	0.558	0.304
				(0.524)	(0.522)	(0.549)	(0.503)	(0.634)
stay-at-home				−13.455	−14.590	−1.563	21.153	4.685
				(23.052)	(23.500)	(18.745)	(14.353)	(39.240)
crime rate					−0.060*			
					(0.035)			
imprison rate					−0.051			
					(0.059)			
poverty rate					−0.819			
					(2.688)			
strong blm						193.394*		
						(113.083)		
week FEs	yes	yes	yes	yes	yes	yes	yes	yes
state FEs	no	yes	yes	no	no	no	no	no
week*black-share FEs	no	no	yes	no	no	no	no	no
N	1,052	1,052	1,052	1,052	1,052	1,052	993	678
R^2	0.422	0.496	0.498	0.456	0.459	0.475	0.488	0.486

Notes: Robust standard errors, are presented in parentheses, stars indicate significance: *, ** and *** indicate statistical significance at the 10-percent level, 5-percent level, and 1-percent level. Specification (2) is our main specification as presented in equation 2.
Additional state-level control variables in specification (5) are derived from d the FBI Uniform Crime Reporting Program. Data are available at `https://eu.usatoday.com/story/money/2020/01/13/most-dangerous-states-in-america-violent-crime-murder-rate/40968963/`
The variable *strong BLM* is equal to 1 if the increase in registered political protest at the beginning of the Black Lives Matter protest was larger than 11, 0 otherwise.
Specification (7) is estimated using model (4) without state-weeks that are strongly affected by the Black Lives Matter protests.
Specification (8) is estimated using model (4) without states that are above-median affected by the Black Lives Matter protests.

Fig 2. Robustness: Effect of negative emotional stress (PCA) on social unrest for different thresholds of unemployment rate and COVID-19 deaths

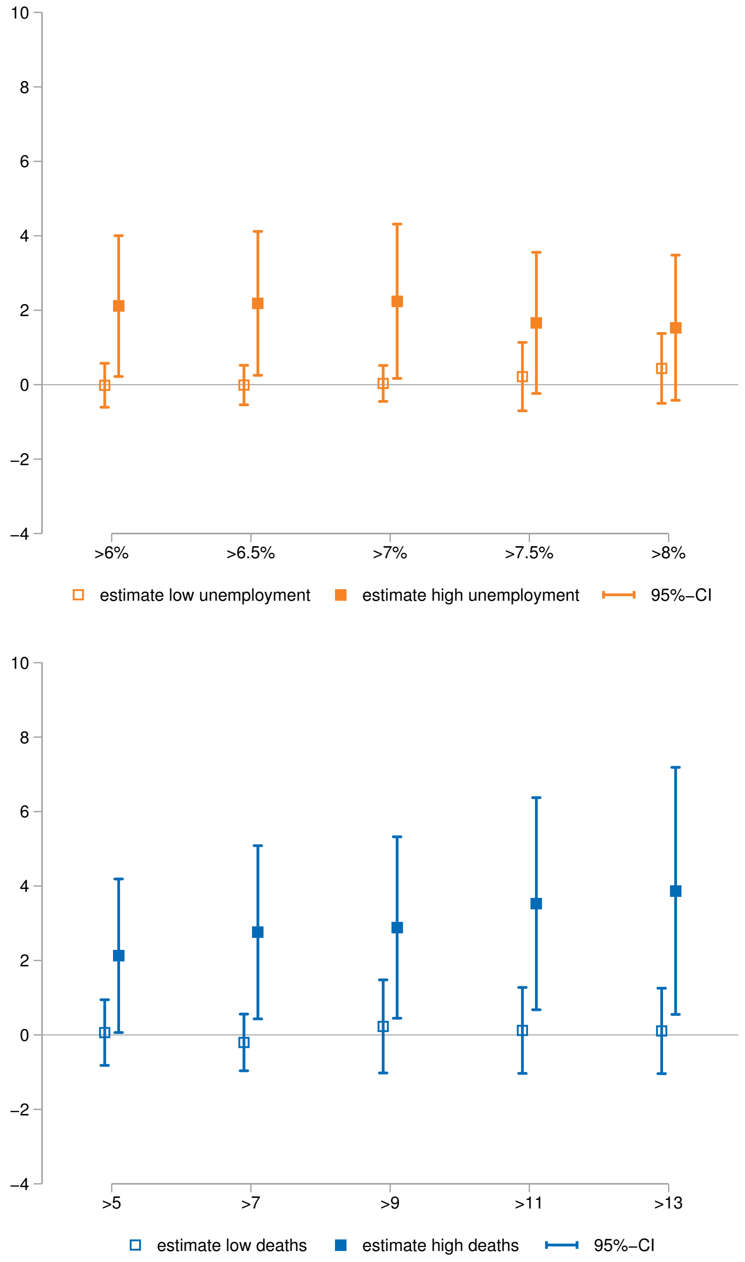

Notes: Estimated coefficients and 95% confidence intervals from estimating model 2 for different thresholds in high/low unemployment. Top: Unemployment. Bottom: Covid-19 deaths.

Fig 3. Robustness: Effect of BAD ECON on social unrest for different thresholds of unemployment rate and COVID-19 deaths

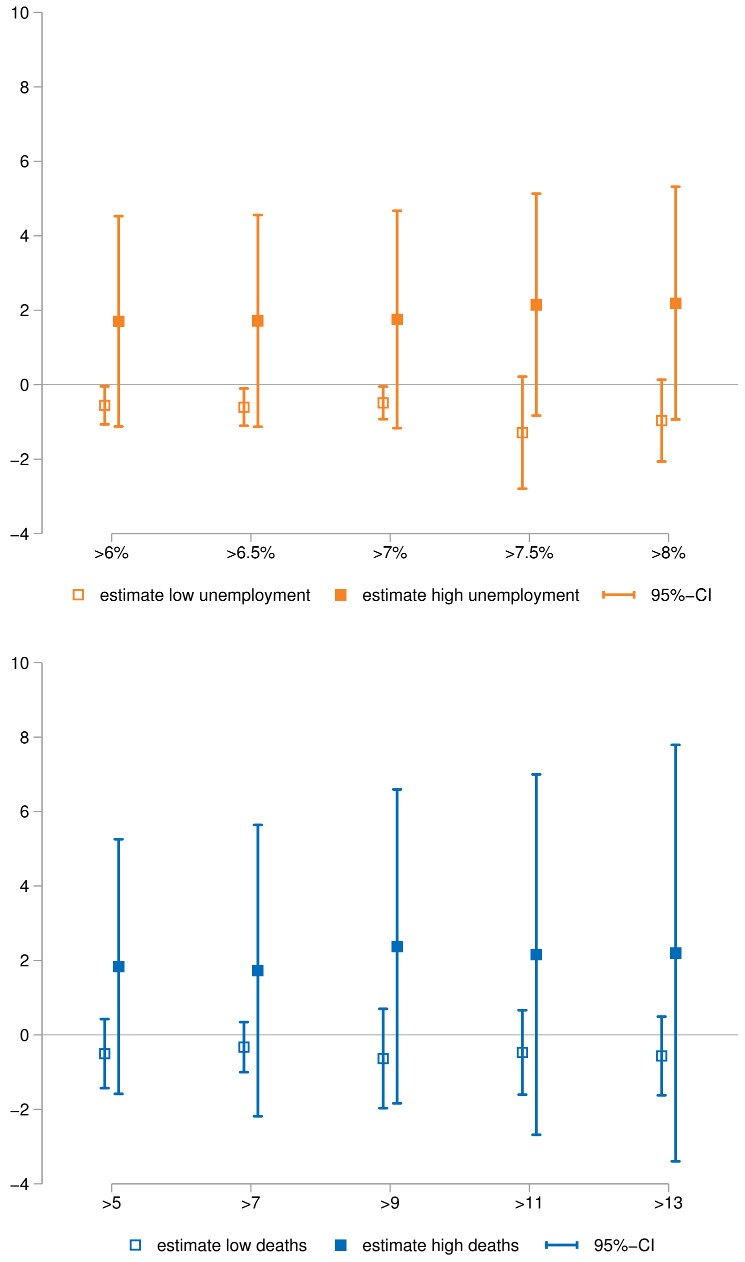

Notes: Estimated coefficients and 95% confidence intervals from estimating model 2 for different thresholds in high/low unemployment. Top: Unemployment. Bottom: Covid-19 deaths.

Fig 4. Negative emotions (PCA) and social unrest (GDELT) - jackknife approach

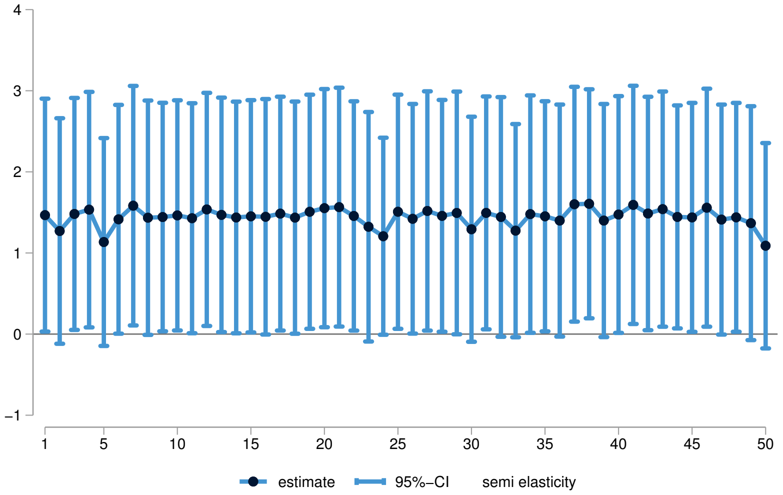

Notes: Estimated coefficients and 95% confidence intervals from estimating model 2 with the sequential omission of 1 state out of 50 US states in our data.

Fig 5. Perception of the economy (BAD ECON) and social unrest (GDELT) - jackknife approach

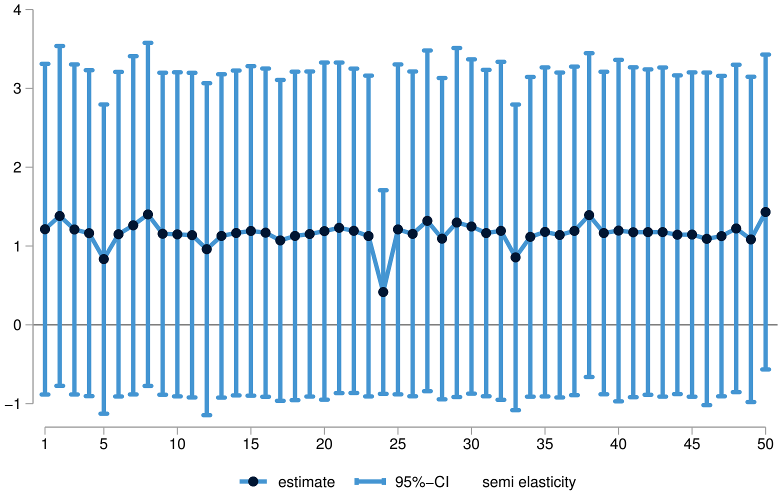

Notes: Estimated coefficients and 95% confidence intervals from estimating model 2 with the sequential omission of 1 state out of 50 US states in our data.
